# Psychiatry and the geriatric syndromes – creating constructive interfaces

**DOI:** 10.1192/pb.bp.115.051649

**Published:** 2017-04

**Authors:** Simon Thacker, Mike Skelton, Rowan Harwood

**Affiliations:** 1 Kingsway Hospital, Derby; 2 The University of Nottingham, Nottingham; 3 Queen's Medical Centre, Nottingham

## Abstract

Integrating mental and physical healthcare is difficult to achieve because of professional and organisational barriers. Psychiatrists recognise the problems resulting from fragmentation of services and want continuity of care for patients, but commissioning and service structures perpetuate these problems. One way forward may be to follow the syndromic model employed by geriatricians as a means of avoiding over-emphasis on diagnosis above the pragmatics of implementing multi-component, coordinated care. Commissioners need to be made aware of the overlap and complementarity of skills possessed by old age psychiatry and geriatric medicine to create joint services for people vulnerable to dementia and delirium. A re-forged alliance between the two specialties will be necessary to turn integrated care for frail, elderly people from rhetoric into reality.

People with severe mental illnesses such as schizophrenia die younger than expected, losing an average 15 years of their lives to cardiovascular and other diseases.^1^ This has led to calls for a more integrated approach to care, with greater emphasis on physical health. In this article, we examine some current issues in the care of older people with severe mental illnesses such as dementia and delirium. We argue that existing systems compartmentalise care and lack the sophistication to deal with the complexity of these illnesses. We suggest that a more collaborative approach between old age psychiatry and geriatric medicine in both hospital and community settings could yield benefits.

Geriatric medicine occupies a position of unfashionable scepticism about the ‘medical model’ of care and its corollary of single-mode interventions. Under the banner of ‘comprehensive geriatric assessment’ attention is paid to function, mental health, and social and environmental factors as well as (usually multiple) acute and chronic medical diagnoses. Elements of prevention, rehabilitation, palliation and mental health models are used flexibly. Care often includes cessation of medicines, recognition of burdens placed on patients by medical intervention, and the prioritisation of comfort, rather than an expectation that death is delayed.^2^ Old age psychiatry can learn from geriatric medicine by embracing complexity, comorbidity and the ultimate inevitability of deterioration, signalling a return of old age psychiatry to its origins.^3^

## What is a geriatric syndrome?

Geriatric syndromes are states of ill health which occur with high frequency in older people but which do not fit into discrete disease categories. They are typically explained by a range of organ dysfunctions and diseases, have functional and social implications, and therefore require care that is complex and traverses traditional disciplinary boundaries. Delirium, falls, immobility and urinary incontinence have been described as geriatric syndromes, but the term is loose and may also include pressure sores and sarcopenia. Dementia and other mental health problems can be thought of in similar terms.^4^

### Dementia as a geriatric syndrome

Dementia is common towards the end of life; 6% of people who die aged 65–69 years and 58% of those who die aged 95 or over have dementia.^5^ The aetiology of late-onset dementia is often multifactorial.^6^ Dementia causes functional decline, and contributes to falls and incontinence. Multi-morbidity is usual^7^ and the symptom burden in the last year of life of people with dementia exceeds that of people dying with cancer.^8^ Dementia causes problems with safety awareness and loss of independence, and is a source of much carer strain. Crises are frequent, and usually have physical, mental, social or care-system contributors. People with dementia frequently transit between home, hospital and care home. These ‘unique and changing personal, psychosocial and physical needs’ are acknowledged in the Memory Services National Accreditation Programme,^9^ but secondary care memory clinics are unlikely to be the only way to address them.^10,11^

A more comprehensive and accessible form of engage – ment is needed, in the form of primary care liaison psychiatry or rapid response community mental health teams (CMHTs). CMHTs for older people led the way in delivering dementia care within communities (long before community geriatrics was a tangible entity).^12^ These teams are under threat, with proposed merger into ‘ageless’ community teams. This would require increasing focus on psychopathology and behaviour, with a likely prioritisation of single (psycho)pathologies. Failure to recognise and appreciate the special needs of patients with dementia and the skills of CMHTs in addressing them risks diminishing patient care.^13^ At the same time, the need to address physical and functional comorbidity suggests that an overt alliance between old age psychiatry and geriatric medicine is needed.

### Delirium as a psychiatric syndrome

Delirium and dementia frequently co-occur in older people in the general hospital. Dementia has achieved a high national profile in the UK. By contrast, delirium barely enters public discourse. Two-thirds of elders acutely admitted to hospital with delirium have underlying dementia^14,15^ and half of people with dementia in acute hospitals have delirium.^16^ Informant history is vital in identifying the two syndromes, but persistent, subacute and subsyndromal delirium is common and the syndromes can be difficult to distinguish, especially where prior dementia was undiagnosed, leading to the coining of the term ‘cognitive spectrum disorders’ to cover both.^17^ Between 10 and 20% of delirium in older people does not have an identifiable precipitant^18^ and the cognitive impact of delirium may persist for months or merge into the onset of a dementia syndrome.^19^ Some survivors develop post-traumatic stress disorder. Uncertainties also exist as to the division of clinical responsibility between geriatricians and psychiatrists. Arguably, neither specialty can do it well in isolation.

The development of health services in the UK over the past 20 years has seen psychiatrists focus on patients in the community while geriatricians have increasingly assumed responsibility for the acute medical intake, militating against collaboration. The diagnostic uncertainties and sequelae of delirium imply the need for systematic follow-up, which geriatric medicine is poorly placed to provide. Psychiatry has a role in working with the third sector and primary care to highlight the malign effects of delirium even in the context of successfully treated acute physical illness.

The publication of delirium guidelines from the National Institute of Health and Care Excellence (NICE) has been a major advance in the promotion of delirium management,^20^ but evidence is weak that any specific intervention or programme of delirium care improves outcomes.^21^ Conversely, there is evidence that multi-component interventions aimed at preventing delirium can reduce its incidence and improve outcomes.^22,23^

Establishing a role for delirium prevention in hospitals and community settings is a challenge given the low profile of delirium training in medical and nursing schools, poor recognition by clinicians, and competing imperatives for healthcare organisations. Conceptualising delirium as both a safety risk to individual patients and an organisational risk due to increased length of stay and adverse health outcomes provides an incentive for change. Old age psychiatrists, with their emphasis on the importance of assessing mental health alongside physical health, can valuably contribute to both delirium care and education.^24^

## Why is delirium prevention not taken more seriously in community psychiatry?

Prevalence studies of delirium in any setting are fraught with the problem of distinguishing delirium from dementia.^25^ A Swedish study of very elderly people found that 52% of people with dementia had experienced delirium within the previous month compared with 5% of those without dementia.^26^ Work in the Netherlands revealed a delirium prevalence of 9% in care homes.^27^

The evidence for the value of delirium prevention programmes in care homes has yet to be established, but there is evidence that coordinated programmes to reduce the prescribing of culprit medications are effective in preventing delirium.^28^ A trial of the effectiveness of multi-component delirium education in preventing the disorder in care homes is underway in the UK.^29^

People with dementia who are living in their own homes and are in receipt of care from CMHTs are also at high risk of delirium. Behavioural and psychological symptoms in dementia (BPSD) are associated with morbid – ities that contribute to delirium, such as falls, nutritional deficits and polypharmacy. Delirium can cause a similar range of symptoms, which may become chronic and constitute BPSD.^30^ Delirium is triggered by physical illness, injury, medications or medication withdrawal, and rightly remains the domain of primary care and geriatricians, but unless psychiatry co-owns the delirium agenda, the cross-over of skills from the management of BPSD will fail to shape delirium prevention, assessment and management. The similarities between delirium prevention programmes and those for the non-pharmacological management of BPSD are striking.^31,32^

## Frailty

Frailty is a state of vulnerability to decline in the face of stressors, in the context of precarious multisystem physiology and social adversity.^33^ Epidemiological evidence highlights the importance of frailty in elderly populations. It has proven difficult to operationalise, but recognisably overlaps with dementia in cross-sectional studies.^34^ One view conceptualises it as the effect of cumulative deficits^35^ and the other as a phenotype characterised by three or more of the following factors: unintentional weight loss, self-reported exhaustion, poor grip strength, slow walking speed and low physical activity.^36^ However, geriatricians recognise that frailty will often manifest through geriatric syndromes – falls, immobility (‘off legs’), delirium (‘more confused’), urinary or faecal incontinence (often associated with delirium, dementia and immobility) and susceptibility to drug side-effects (antidepressants making a patient ‘very drowsy’).^37^ The maturation of old age psychiatry as a specialty has been facilitated by the advent of specific therapies for Alzheimer's disease, but an emphasis on prescribing for Alzheimer's disease has selected against attendance by frail elders and those with non-Alzheimer pathology (particularly vascular dementia) and cognitive deficits outwith a full-blown dementia syndrome.

Comprehensive geriatric assessment aims to consider the full range of factors contributing towards frailty (Table 1). Its implementation is linked to better outcomes.^38^ It has parallels with biopsychosocial assessment, although this does not have similar evidential weight behind it, and indeed has been criticised by the psychiatric profession as ‘mere eclecticism’.^39^ The accusation of vagueness laid against biopsychosocial assessment suggests that it needs to find a home within an operationally defined, evidence-based structure such as comprehensive geriatric assessment. The two processes differ only in the emphasis and differential expertise of the clinicians undertaking them. Geriatricians recognise that they tend to neglect the mental health dimension, which may be reduced to a brief cognitive assessment or screening test for depression. There is therefore a fertile opportunity for mutual learning between geriatrics and psychiatry in the area of the assessment of frailty.

**Table 1:** Components of comprehensive geriatric assessment

Domains	Items to be assessed
Medical	Co-morbid conditions and disease severity
	Medication review
	Nutritional status
	Problem list

Mental heath	Cognition
	Mood and anxiety
	Fears

Functional capacity	Basic activities of daily living
	Gait and balance
	Activity/exercise status
	Instrumental activities of daily living

Social circumstances	Informal support available from family or friends
	Social network such as visitors or daytime activities
	Eligibility for being offered care resources

Environment	Home comforts, facilities and safety
	Use or potential use of telehealth technology etc.
	Transport facilities
	Accessibility to local resources

Reproduced from Martin, 2010.^40^

## Sustainable integration

The needs of older people with mental health disorders are not well served by a retreat to psychiatric specialisation, restrictive referral criteria or commissioning models based on activity alone. Geriatric medicine recognises and embraces complexity and uncertainty, and responds by flexibly utilising a variety of models, albeit at times with tensions between them. Old age psychiatry can learn from comprehensive geriatric assessment. Geriatric medicine needs to take mental health more seriously, to increase the depth and sophistication with which it assesses the mental state, and can learn from person-centred care and recovery models. The logical future for both disciplines is in collaboration and integration that transcends organisational and cultural barriers.

A sustainable integration of physical and mental healthcare for older people will require more than cooperation between clinicians. The joining of forces between psychiatrists and geriatricians can take place at an organisational level when mental health and community trusts merge. However, we also need a flexible, accessible, consultative model of psychiatry that seeks to empower a broad range of community practitioners and will be the means to generate influence on problems that are just too common and multi-morbid to be addressed solely by clinic-based approaches or a single professional discipline. This reinforces the need for CMHTs for older people, and highlights the need to change the commissioning model from one based on activity defined by clinic attendances. Crises in care homes, for example, often represent a complex interplay of medical, mental, social and environmental issues best addressed by timely multi-disciplinary input rather than transferring responsibility on to a single discipline. Accessible advice on a broad range of cases managed primarily by other teams (‘liaisons’) allows for teaching and upskilling while preserving specialty provision for patients with more severe, less tractable mental health problems. These teaching and support roles need a commissioning model.

CMHTs are in a good position to identify dementia and promote delirium prevention measures. Working alongside community geriatrics will strengthen old age psychiatry by allying it with the developing evidence base and increasing its workforce.

The Rapid Assessment Interface and Discharge (RAID) model developed in Birmingham has captured the attention of policy-makers through its widely publicised potential financial savings. But it also demonstrates the total immersion of mental health practitioners in the multi-professional melee that is acute hospital care.^41^ Why not import this style of working into primary care and scale down the centralised psychiatric clinic? This has been tried in Gnosall, Staffordshire, where a model of primary care liaison psychiatry has created a well-received, effective service for people with dementia.^42^

## Conclusion

Psychiatrists must lobby commissioners to recognise the plight of those frail, elderly patients who are not living well but dying with the multiple comorbidities of dementia within a healthcare system that fails to accommodate complexity. People with dementia are prone to crisis and comorbidity, necessitating attention to physical health (parity of esteem) equal to that developing for other severe mental illness. Emphasising geriatric syndromes (and the importance of sound mental health assessment within comprehensive geriatric assessment) is a good way to defend old age psychiatry while at the same time developing integrated physical and mental healthcare for older people. An invigorated liaison psychiatry, underpinned by a re-forged alliance between old age psychiatry and geriatric medicine, gives a pointer to how integration might work, and enables parity of esteem for mental and physical health.
